# Clinical Research of Traditional Chinese Medicine Needs to Develop Its Own System of Core Outcome Sets

**DOI:** 10.1155/2013/202703

**Published:** 2013-11-07

**Authors:** Li Zhang, Junhua Zhang, Jing Chen, Dongmei Xing, Wei Mu, Jiaying Wang, Hongcai Shang

**Affiliations:** Center for Evidence-Based Medicine, Tianjin University of Traditional Chinese Medicine, 312 Anshanxi Road, Nankai District, Tianjin 300193, China

## Abstract

Currently, quality issues concerning clinical research of traditional Chinese medicine (TCM) have come into the spotlight. It has been recognized that poorly-devised research methodology largely restricted the development of clinical research in TCM. The choice of appropriate outcome measurements is key to the success of clinical research; however, the current procedure for outcomes selection in clinical research of TCM is problematic due to the underdevelopment of clinical methodology. Under this circumstance, we propose the introduction to the concept of Core Outcome Set (COS) and discuss the feasibility of developing a COS system that caters for clinical studies in TCM, in the hope that the outcome evaluation system could be up to international standards.

## 1. Introduction

Clinical effectiveness is not only important for traditional Chinese medicine (TCM), but for all medical systems. Clinical research in TCM has been developed relatively later. Largely due to the many existing methodological defects of TCM trials, the efficacy of TCM remains controversial [1, 2]. All these issues have created a bottleneck in the development of TCM. There is a growing recognition that more attention has been paid to enhance the quality in clinical research of TCM [3, 4]. Especially in this decade, a new discipline, clinical evaluation of TCM, has formed into being and its development has been supported by the Chinese Major Science and Technology Projects. Owing to concerted efforts from a panel of experts with multidisciplinary background, the quality of clinical research of TCM has been improved, in terms of the construction of clinical research platform and the introduction of process management. There have been several achievements, for instance, the construction and implementation of the central randomization system, the application of the clinical data management system, the introduction of ethical review and trial registration, the promotion of the Consolidated Standards of Reporting Trials (CONSORT) statement, and the standardization of the data processing and analysis techniques. 

## 2. Problems with Outcome Measures in Clinical Trials of TCM

Consensus on regarding what constitute a good design has been reached by most scholars, except some characteristics of TCM which have unique difficulties, including placebo making, sham control of acupuncture, tailoring treatment principle, and diagnostic system. Outcome measure is one of the key factors. For example, a systematic review of 35 trials investigating the efficacy of TCM for chronic obstructive pulmonary diseases (COPD) reported a total of 22 different outcomes [5]. Another systematic review [6] including 17 randomized controlled trials (RCTs) with compound salvia dropping pills for unstable angina pectoris reported 11 outcome measures. Among all included studies, 11 studies (65%) reported angina improvement, 10 reported electrocardiogram improvement, and only one study reported the incidence of major cardiovascular events. 15 trials (88%) had a treatment duration of 4 weeks, too short to identify clinically significant improvement on angina. A systematic review [7] of primary studies on a combination of TCM treatment regimens for improving the movement function of poststroke patients included 34 RCTs with 11 different outcome measures, all being intermediate outcomes. 29 (85%) RCTs reported total effective rate; however, the original data and the standard against which to judge efficacy were not found in the primary study. In 23 RCTs (85%), the treatment period lasted less than 30 days. Moreover, only one study reported adverse events.

Problems concerning the choice and reporting of outcome measures for clinical trials of TCM were summarized as follows [5, 8–10]. (1) Outcome measured and reported in clinical trials of the same condition varied greatly, and there was apparent selective reporting bias. (2) Soft outcome measures are often employed such as percentage of patients perceiving benefit. (3) Subjective outcome measures were in a dominant position while objective outcome measures were less frequently adopted. (4) Lack of agreed and standardized evaluation criteria for TCM-related outcomes (e.g., tongue and pulses). (5) Surrogate endpoints bearing limited importance to practitioners and patients, such as biochemical indicators, were widely adopted, whereas important endpoint outcomes were less frequently reported. (6) Insufficient attention has been paid to the reporting of adverse event and adverse drug reaction associated with the use of herbal medicines. 

## 3. Origin and Development of a Core Outcome Set (COS)

Concerning the same health care issue, the prospective of the patients, clinicians, researchers, and policymakers may vary greatly. Lack of consideration of this issue will lead to study results with less practical value. Therefore, selecting appropriate outcome(s) is crucial in the design of clinical trials. In addition, the results of systematic reviews and meta-analysis are an important source of evidence for health-related decision making, and the credibility of these results are based on the validity and quality of primary studies. If there are defections in the design and reporting of individual trials, systematic reviews and meta-analysis will not generate reliable evidence. For instance, the results of several studies cannot be compared and combined as a result of heterogeneity across studies, thus leaving valuable information wasted and the quality of evidence less rigorous [11].

The minimum outcome set which should be measured in a clinical trial for a specific disease, namely, a core outcome set (COS) [12], is an effective way to solve the above issues. It can simplify the design of clinical trials, reduce the risk of selecting inappropriate outcomes and minimize outcome reporting bias [13]. More importantly, the use of COSs can reduce the heterogeneity of the reported outcomes between studies, so that the results of different trials can be compared and combined [14].

As early as the late 1870s, the World Health Organization (WHO) has put forward the notion of adopting standardized outcomes and has compiled a handbook [15]. Since 1992, the OMERACT (Outcome Measures for Rheumatology Clinical Trials) collaboration has been committed to advocate and develop the COSs for clinical trials in rheumatology [16] and has made significant contributions [17]. Since 2002, the IMMPACT (Initiative on Methods, Measurement, and Pain Assessment in Clinical Trials) group has started working on COSs in trials with treatment for pain [18]. To date, more than 50 organizations or groups devoted to COS have been founded, involving several conditions and diseases such as eczema [19] and wound [20]. 

The projects for establishing and promoting COSs is gradually underway in different areas; however, the work has not gained worldwide popularity. Internationally acknowledged COSs has been sparse and a general guideline for COSs development is lacking [21]. Hence, clinical methodologists have launched the COMET (Core Outcome Measures in Effectiveness Trials) Initiative [22].

As an international academic organization, the initiatives gathered experts from different areas who are interested in promoting the development of COSs. Its management group is composed of four well-known experts in methodological research, including professor Doug Altman from the Oxford University, Professor Mike Clarke (the former director of the UK Cochrane Center) from the Queen's University in Belfast, Professor Paula Williamson from the University of Liverpool, and Professor Jane Blazeby from the University of Bristol [23]. The first COMET meeting was held in Liverpool in 2010 with a total of 110 participants including clinical researchers, systematic reviewers, clinicians, editors, health care consumers, trial funders, policy makers, and trials registries and regulators. The experts reached a consensus that it was necessary and urgent to promote COMET, and the relevant work should be conducted immediately. In July 2011, the COMET group held the second meeting, emphasizing the importance of developing COSs and the coordination and integration of information from various areas of health care, as well as the need to promote the development and application of COSs [24]. The third meeting will focus on how to select, evaluate, and use measurement of core outcomes to further promote the progress of relevant work [25].

## 4. Why COSs in Clinical Trials of TCM Are Established?

The issues surrounding outcome measures in clinical trials of TCM have been discussed widely. However, the focus was on the method of categorization and the choice of expressions for the outcomes. What has also been emphasized is that the chosen outcomes should represent the characteristics of TCM, for example, outcome measurements relevant to the TCM syndrome and other soft endpoints [26]. The above ideas on constructing COSs in the field of TCM have not aroused sufficient attention. During the recent decade, clinical studies with TCM have mushroomed and the quality has also improved. However, the majority of these studies have been rejected for publication on leading international medical journals, and their findings also received few citations and little recognition. The lack of a uniform standard for selecting and reporting outcomes may be one of the factors devaluing the clinical research of TCM. As a result, promoting methodological research on COSs and establishing COSs based on the unique features of TCM are good solutions. It can also improve the quality of clinical research of TCM and the practicability of study results.

Furthermore, the development of clinical methodology for TCM in China kept following international trends, rather than taking initiatives. For instance, the reporting standards of herbal interventions were established by a group of Canadian experts [27], and the reporting standards in clinical trials of acupuncture were set by a panel of western scholars [28]. Neither of them involved a TCM specialist. It is time to take initiatives in establishing COSs catering for TCM clinical research in order to facilitate the work of scientific and rational evaluation of clinical efficacy of TCM.

At the time when the development of COSs in clinical research has just been started internationally, TCM scholars shall seize the opportunity and devise key techniques and methods for establishing COSs for TCM clinical research. In this way our research methodology may meet international standards. And we believe the uniqueness of TCM can add to the variety and vitality and also expand the breadth and depth of international COS research. 

## 5. The Feasibility of Establishing COS in Clinical Trials of TCM

The COMET initiative team, the OMERACT working group, and the WHO have conducted extensive research on the development and application of COSs and have accumulated valuable experiences. The practical knowledge of and experiences with organization and management of the COMET initiative team will be of particular value for the development of COSs in clinical research of TCM. The currently existing methodological guidelines can provide guidance on our work. With advances in clinical epidemiology and evidence-based medicine, the development of research methodology and its application in clinical studies of TCM have made encouraging progress. Several multidisciplinary research teams have been formed, including scholars specialized in TCM, clinical epidemiology, evidence-based medicine, clinical pharmacology, statistics, and bioinformatics. This provides the professional research team necessary for establishing COSs in clinical trials of TCM. 

The detailed work will be done based on guidance on developing the COMET initiative. Firstly, several diseases on which TCM therapies have unique effects should be identified such as chronic noncommunicable diseases. Secondly, systematic reviews especially high-quality Cochrane reviews for these diseases will be selected to determine outcomes related to specific disease. Thirdly, information from patients and TCM clinicians will be collected by using consensus methods such as the Delphi technique. Finally, outcome measures from systematic reviews and the consensus will be integrated.

The development of COSs in the field of TCM research will help improve the design and conducting of TCM trials to international standards, thereby lending credibility to the results.

## References

[1] Zhang JH, Shang HC, Dai GH (2007). Key points of protocol design on clinical therapeutic research of traditional Chinese medicine. *Chinese Journal of Integrated Traditional and Western Medicine*.

[2] Di MY, Tang JL (2013). Adaption and application of the four phase trials to traditional Chinese medicines. *Evidence-Based Complementary and Alternative Medicine*.

[3] Shang HC, Zhang BL (2010). To improve the overall level of traditional Chinese medicine research by goverment leading and multiple participation. *Chinese Journal of Evidence-Based Medicine*.

[4] Shang HC, Zhang BL, Wang YY (2013). Clinical research of traditional Chinese medicine needs ‘a breakthrough’. *Tianjin Journal of Traditional Chinese Medicine*.

[5] Wang MH, Li JS, Yu XQ (2011). A systematic review of outcomes of randomized controlled trials about stable-stage of chronic obstructive pulmonary disease. *Chinese Journal of Gerontology*.

[6] Zhang JH, Shang HC, Gao XM (2008). Compound salvia droplet pill, a traditional Chinese medicine, for the treatment of unstable angina pectoris: a systematic review. *Medical Science Monitor*.

[7] Junhua Z, Menniti-Ippolito F, Xiumei G (2009). Complex traditional Chinese medicine for poststroke motor dysfunction: a systematic review. *Stroke*.

[8] Ernst E (2006). Methodological aspects of traditional Chinese medicine (TCM). *Annals of the Academy of Medicine Singapore*.

[9] Chen RQ, Wong CM, Lam TH (2012). Construction of a traditional Chinese medicine syndrome-specific outcome measure: the Kidney Deficiency Syndrome Questionnaire (KDSQ). *BMC Complementary and Alternative Medicine*.

[10] Wu TX, Shang HC, Bian ZX (2010). Recommendations for reporting adverse drug reactions and adverse events of traditional Chinese medicine. *Chinese Journal of Evidence-Based Medicine*.

[11] Tovey D (2010). *The Impact of Cochrane Reviews*.

[12] Clarke M (2007). Standardising outcomes for clinical trials and systematic reviews. *Trials*.

[13] Williamson PR, Altman DG, Blazeby JM, Clark M, Gargon E (2011). The COMET (core outcome measures in effectiveness trials) initiative. *Trials*.

[14] Williamson PR, Altman DG, Blazeby JM (2012). Developing core outcome sets for clinical trials: issues to consider. *Trials*.

[15] World Health Organization (1979). *WHO Handbook for Reporting Results of Cancer Treatment*.

[16] Tugwell P, Boers M (1993). OMERACT conference on outcome measures in rheumatoid clinical trials: introduction. *Journal of Rheumatology*.

[17] Tugwell P, Boers M, Brooks P, Simon L, Strand V, Idzerda L (2007). OMERACT: an international initiative to improve outcome measurement in rheumatology. *Trials*.

[18] Dworkin RH, Turk DC, Farrar JT (2005). Core outcome measures for chronic pain clinical trials: IMMPACT recommendations. *Pain*.

[19] Schmitt J, Williams H (2010). Harmonising outcome measures for eczema (HOME). Report from the first international consensus meeting (HOME 1), 24 July 2010, Munich, Germany. *The British Journal of Dermatology*.

[20] Gottrup F, Apelqvist J, Price P (2010). Outcomes in controlled and comparative studies on nonhealing wounds: recommendations to improve the quality of evidence in wound management. *Journal of Wound Care*.

[21] Sinha IP, Smyth RL, Williamson PR (2011). Using the Delphi technique to determine which outcomes to measure in clinical trials: recommendations for the future based on a systematic review of existing studies. *PLoS Medicine*.

[22] http://www.comet-initiative.org/.

[23] http://www.comet-initiative.org/about/whoweare.

[24] http://www.comet-initiative.org/about/overview.

[25] http://www.comet-initiative.org/meeting.

[26] Lai SL (2002). Key points about clinical effectiveness assessment of traditional Chinese medicine. *Journal of Guangzhou University of Traditional Chinese Medicine*.

[27] Gagnier JJ, Boon H, Rochon P, Moher D, Barnes J, Bombardier C (2006). Reporting randomized, controlled trials of herbal interventions: an elaborated CONSORT statement. *Annals of Internal Medicine*.

[28] MacPherson H, Altman DG, Hammerschlag R (2010). Revised STandards for Reporting Interventions in Clinical Trials of Acupuncture (STRICTA): extending the CONSORT statement. *PLOS Medicine*.

